# Supplementary material for: Rice Seed Protrusion Quantitative Trait Loci Mapping through Genome-Wide Association Study
Source: Plants (Basel). 2024 Jan 3;13(1):134. doi: 10.3390/plants13010134 (PMC10780921; doi:10.3390/plants13010134)
Supplement: Supplementary file 1 [file plants-13-00134-s001.zip › Supplementary Figure S1. Temporal expression pattern of PP-associated genes in seed and seed aleurone. .pdf]

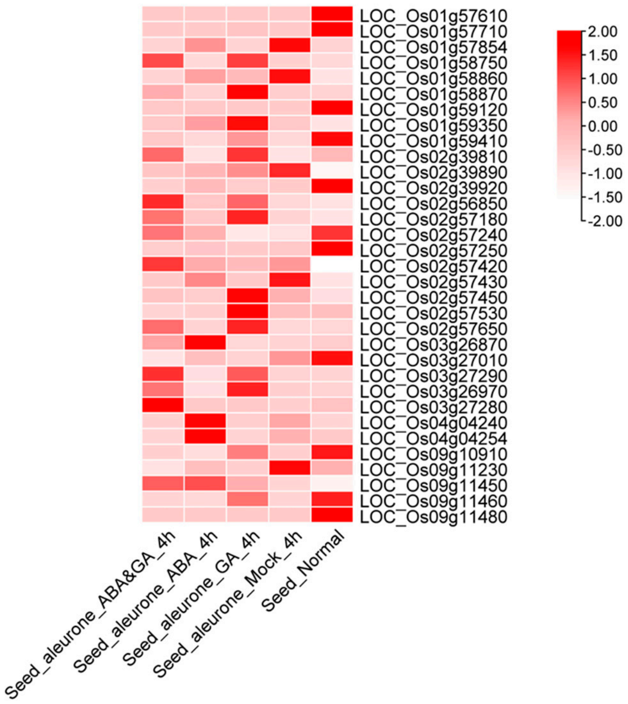

Figure S1. Temporal expression pattern of PP-associated genes in seed and seed aleurone. Expression data in seed and seed aleurone were downloaded from Rice Expression Database website (http://expression.ic4r.org/search). The heatmaps represented hierarchical clustering of relative expression levels of candidate genes in seed, seed aleurone and aleurone induced 4h by ABA&GA, ABA, and GA. The scale for relative expression levels (after normalized by z-score) is denoted by color bars, with red representing the high expression levels, pink representing the medium expression and white representing the low expression.
